# CASEPLUS-SimPat: An Intersectoral Web-Based Case Management System for Multimorbid Dementia Patients

**DOI:** 10.1007/s10916-020-1533-9

**Published:** 2020-02-08

**Authors:** Bianca Steiner, Bettina Zippel-Schultz, Andrea Popa, Nils Hellrung, Stefan Szczesny, Claudia Möller, Carsten Schultz, Reinhold Haux

**Affiliations:** 1 Peter L. Reichertz Institute for Medical Informatics of TU Braunschweig and Hannover Medical School, Mühlenpfordtstr. 23, 38106 Braunschweig, Germany; 2 German Foundation for the Chronically Ill, Berlin, Germany; 3 Department of Technology Management, Institute of Innovation Research, University of Kiel, Kiel, Germany; 4 symeda GmbH, Braunschweig, Germany; 5 AGAPLESION gAG, Frankfurt am Main, Germany

**Keywords:** Dementia, Case management, Intersectoral collaboration, Web-based

## Abstract

One main challenge of health care systems in future will be the care and treatment of dementia patients. To their advanced age, dementia patients do not only have limitations in cognition, perception, and articulation, but also suffer from several other diseases (multimorbidity). These patients have sophisticated needs for assistance, care, and treatment relying on different health care sectors. Well-known communication and coordination deficits between sectors are intensified in the case of dementia, to the above described limitations. Coordination processes concerning the transition of patients from hospitals to inpatient or outpatient aftercare pose particularly difficult. To show a possible future direction for improving intersectoral care of dementia patients, we developed the web-based case management system CASEPLUS-SimPat as part of the project SimPat “Securing Integrated Care for Multi-morbid Patients with Dementia using an IT-based Service Concept”. CASEPLUS-SimPat allows health care professionals, hospital employees and general practitioners, to jointly access patient data and exchange information about the treatment of patients. The case management system was implemented in a three-tier architecture with a role-based authorization concept. A portal for relatives complements the system. By providing precise information and e-learning services, caring relatives are integrated into the care process. In a pilot test, CASEPLUS-SimPat shows a good usability. Adapting aspects, such as keywords used to structure entries will make it easier to find required information, and thus will increase usability. Next steps comprise integration of further health care professionals and care facilities as well as real-life testing in terms of feasibility and effectiveness.

**Electronic supplementary material:**

The online version of this article (10.1007/s10916-020-1533-9) contains supplementary material, which is available to authorized users.

## Introduction

Integrating care can effectively improve care quality and outcomes while at the same time prudently managing scarce resources in the health care sector [1]. Case management is one of the techniques used to improve care integration across sectors. Particularly in complex care situations, such as those of multimorbid patients with dementia, case management may play an important role in managing demanding health care situations, and help health care professionals to provide comprehensively care processes for their patients [2]. Unfortunately, research on the effectiveness and best-practices to implement case management in the context of dementia patients remains sparse, with a gap especially addressing interventions at a service and systems level [2]. This is partly due to the complications of dementia itself, which increasingly debilitates patients during its progression [3]. However, patients with dementia are more likely hospitalized for acute illnesses, such as fractures due to falls, infections or complications of chronic diseases [2]. Particular coordination problems arise in the post-operative care of patients with fractures, as various medical specialties, therapists, nurses and the home environment have to be integrated into the care.

The collaborative project “Securing Integrated Care for Multi-morbid Patients with Dementia using an IT-based Service Concept” (SimPat) addresses the challenges of integrated care in the discharge process of dementia patients. It aims to develop, implement and evaluate a computerized case management system for these patients. Through the use of information and communication technologies (ICT), caregiving relatives, professional caregivers as well as physicians shall be better connected. Thus, all those involved in care should be provided with relevant information beyond institutions in an appropriate form (patient-centered care). Coordination processes concerning the transition of patients from hospitals to inpatient or outpatient aftercare are particularly challenging (discharge management). Therefore, the digital case management system needs to support those processes, by integrating and connecting all actors involved. The case management system involves hospital employees (inpatient setting), family doctors that secure the treatment after the hospital stay (outpatient setting), and caregiving relatives (homecare setting) in the cross-sectoral integration of health care professionals.

### Objectives

The general objective of this research is to show a possible future direction for improving intersectoral care of dementia patients, exemplified by discharge management processes. A digital, interoperable case management system will be used to facilitate a coordinated information exchange between inpatient and outpatient health care professionals as well as caregiving relatives.

This paper focuses on the presentation and pilot evaluation in terms of usability of the case management system CASEPLUS-SimPat with regard to the software architecture and its basic functionalities.

### Related Work

Case management is well-known as “*[…] a collaborative process of assessment, planning, facilitation and advocacy for options and services to meet an individual’s holistic needs through communication and available resources to promote quality and cost-effective outcomes*” [4]. There are some approaches to case management in dementia. Fields of application range from (a) assistance with support services to (b) action plans for caregivers to (c) guidance for family doctors [5]. Nurses, social workers and mental health workers fulfil the coordination role of the case manager [5]. The case management methods presented so far are often supported by ICT, but mainly focus the services of case managers. Other ICT solutions focus on the empowerment of relatives or patients. Only a small number of approaches exist that support the integration of the inpatient and outpatient care-setting, and even a smaller number integrate the caregiving relatives into the care process, linking case management with personal environments of patients [6]. To the authors’ knowledge, there is no digital case management system to support this crucial communication and coordination process between hospital employees and general practitioners with focus on discharge management of multimorbid dementia patients, integrating the caregiving relatives as part of the process.

## Material and Methods

### Setting

To achieve the aims of SimPat, we analyzed the status quo of care focusing on care processes of dementia patients following traumatic falls. First, we identified key processes and deduced information and communication gaps [7]. Second, we determined both professional and informal caregivers needs by qualitative interviews. The analyses revealed several information and communication gaps as well as a variety of stakeholders needs in the discharge management of dementia patients following traumatic falls. There are several barriers to clinical integration – structural, technical, and process related. One major issue is the fact that healthcare providers often have little or no joint knowledge about their patients due to existing functional silos, missing integrated information systems and health care processes, and diffused organizational and legal responsibilities [7]. Information about the patient’s medical history, social environment, and current treatment is often incomplete [7]. Based on these results, the digital case management system CASEPLUS-SimPat was implemented.

Pilot site for CASEPLUS-SimPat is the region of Darmstadt, Germany. AGAPLESION ELISABETHENSTIFT (EKE) is the second-largest hospital in this region and serves as starting point for the case management system [8]. To its interdisciplinary treatment concept, EKE is particularly well suited for geriatric patients [8]. The observation period per patient with CASEPLUS-SimPat (episode of care) encompasses the admission of patients to EKE to discharge and subsequent nursing and medical aftercare up to three weeks after discharge.

### Software Development

CASEPLUS-SimPat bases on the software product CASEPLUS by symeda GmbH. CASEPLUS is a web-based platform for integrated health care that has been used in the field of prevention and treatment of psychiatric diseases for some time. We built on the results of the process and requirements analyses [7] and adapted this basic software to the use case *intersectoral case management of multimorbid dementia patients*. This included, among other things, the implementation of HL7 interfaces, enabling CASEPLUS-SimPat to be used as complementary system to ORBIS, so that manual double entries are avoided. According to the different professions within the care process of dementia patients, the authorization concept was customized by adding new roles and permissions. Moreover, a network of care was introduced to list professional and informal caregivers involved in the care process. This network also clarifies the function of a caregiver within the process, such as the role of the legal guardian.

## Software Description

CASEPLUS-SimPat is a web-based platform for supporting discharge management for multimorbid dementia patients, especially those with fractures. The system can be accessed from a compatible web browser.^1^ Available patient data can be imported directly from ORBIS, the hospital and clinical information system (HIS/CIS), and is made available to all authorized persons. The primary aim is to support the communication between hospital employees (e.g. doctors, nurses, discharge managers) and family doctors to ensure the availability of accurate, extensive patient information across organizational boundaries. To integrate relatives more closely into the care process, CASEPLUS-SimPat is supplemented by a portal, especially designed for caregiving relatives. In this way, relatives can receive assistance through e-learnings for relevant questions.

### Software Architecture

**Fig. 1:**
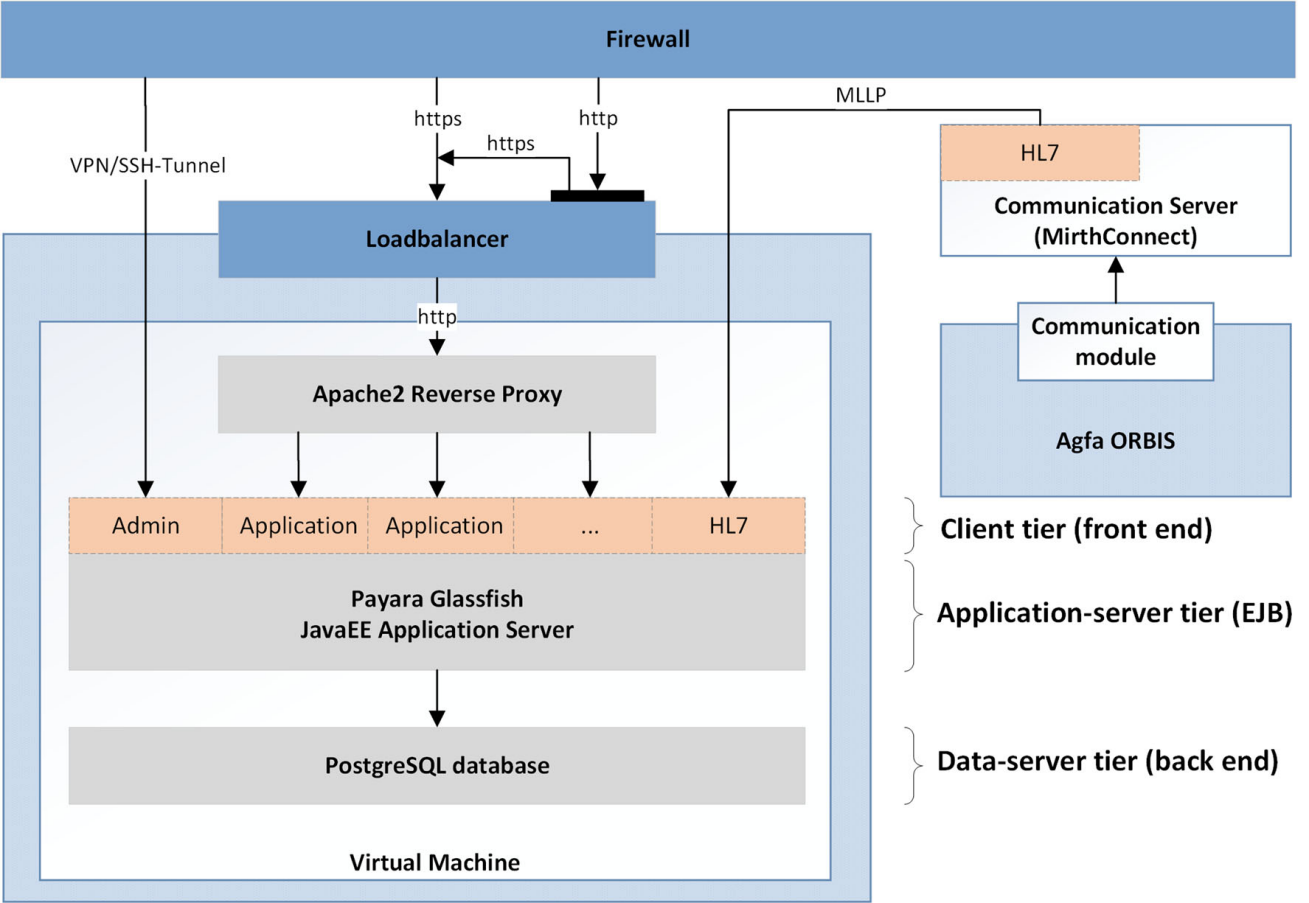
CASEPLUS-SimPat system architecture (grey = daemon; orange = application programming interface; dark blue = service; light blue = application system; white = virtual machine)

CASEPLUS-SimPat is a web-application based on a Java platform, Enterprise Edition, implemented in a three-tier architecture behind a firewall (Fig. 1). Different modules are arranged on individual layers, in which the functions of the system are implemented logically separated from each other. Individual modules can communicate with each other via defined interfaces.

Users interact with CASEPLUS-SimPat via a web-browser through an https encrypted connection. Thus, access is only granted to individual web or user interface (UI) elements. Furthermore, an implementation according to Java Servlet specifications as a Web Application Archive enables distributed access to individual UI elements. Depending on the authorizations allocated to their role, only certain UI applications for navigation through the system are displayed to a logged-in user.

#### Interfaces

The case management system functions as secondary system obtaining patient data – master data, case data, treatment data – from ORBIS (Fig. 1). CASEPLUS-SimPat is linked to the ORBIS infrastructure via a MirthConnect communication server. This server is used for any communication between ORBIS and external application systems within the hospital, including ORBIS’ own developed and commercial application systems. For data exchange, several HL7 interfaces were implemented for ORBIS, MirthConnect communication server as well as CASEPLUS-SimPat. Using a Minimal Lower Layer Protocol (MLLP), HL7 messages (only relevant segments) can be transmitted via TCP/IP from ORBIS to CASEPLUS-SimPat (unilateral communication). Here, the HL7 Admit Discharge Transfer (ADT) and the Medical Document Management (MDM) message types are used. This enables the communication of patient demographics, e.g. name, address, admission date, or patient identification number, and the export/import of entire documents, e.g. the doctors letter or medication plan. Depending on the configuration of an individual HL7 segment, the transmission of respective data to CASEPLUS-SimPat will be done as soon as a corresponding trigger event has been activated. For example, the discharge date of a patient will not be transferred to CASEPLUS-SimPat as long as the corresponding ADT flag “final discharge date” is not set in ORBIS.

#### Access and Authorization Concept

CASEPLUS-SimPat was implemented on a role-based authorization concept. Hence, users can only view tabs and use functions according to their assigned role. Also, a user can only access the data of a patient whose treatment he is involved in. For this purpose, each user is initially assigned to a role by the user administrator. Thereby, the following roles are distinguished: (a) discharge manager for hospital employees, (b) practitioner for general practitioners, (c) case manager, and (d) relative.

To give a health care professional or relative initial access to a patient’s case file, a two-factor authentication is used. The roles “discharge manager” and “case manager” can generate a unique, patient specific authorization number. This token is printed out and handed over to the yet unregistered caregiver by the patient or a patient’s legal guardian. This approach ensures the patient’s right to freely select physicians. In combination with the health insurance number of the patient, this number can be redeemed to gain access to a patient’s case file, provided that there is a login to CASEPLUS-SimPat.

### Modules

Three types of modules are distinguished: (1) basic modules, (2) information provision modules, and (3) modules of the portal for caregiving relatives.

#### Basic Modules

Two basic modules provide essential structures for information provision: the *address book* and the *workspace*.

The *address book* is used for administrations of possible organizations and persons involved in the care process of dementia patients. It does not summarize the network of one patient. Here, all organizations and professional health care providers, participating in SimPat are stored in two listings. Each organization listing includes the name and type (hospital, nursing home, doctor’s practice) as well as contact details. Additionally, a health care professional list summarizes information on individual staff members of an organization, such as the medical specialization and a reference to the contact details of the facility they belong to.

**Fig. 2:**
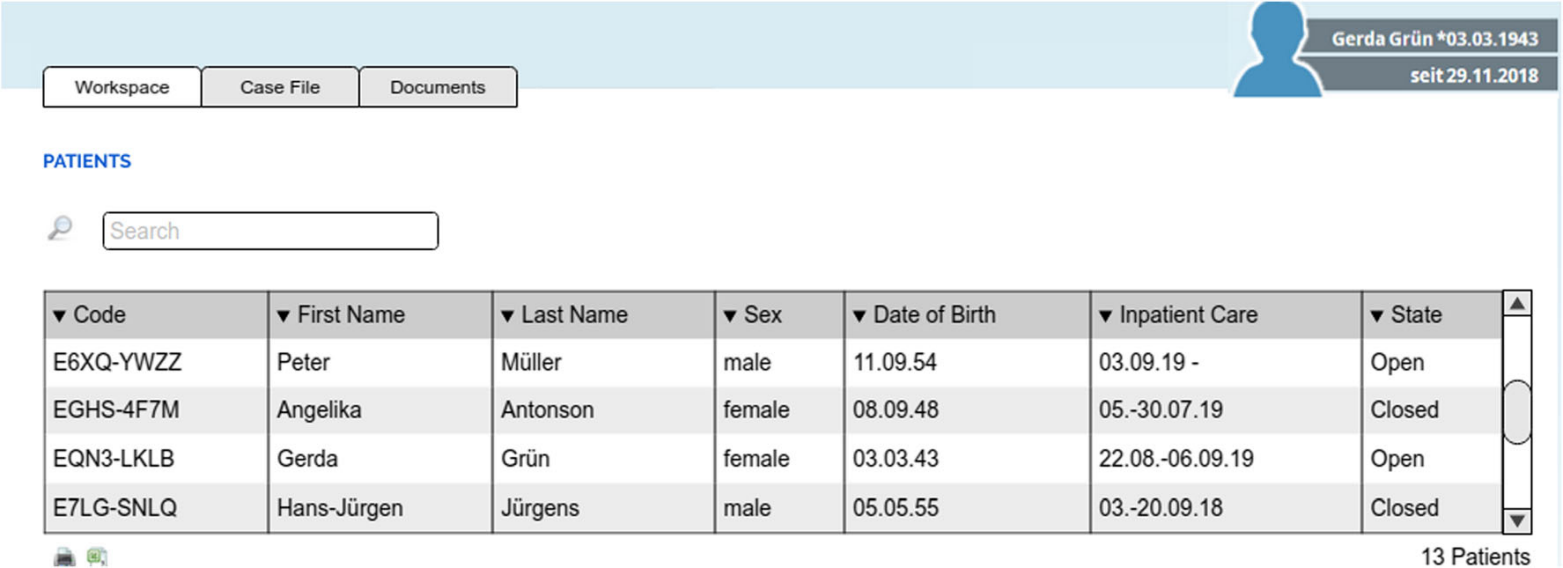
CASEPLUS-SimPat MockUp – Workspace (list of patients). (For better understanding a MockUp instead of a screenshot is used, as CASEPLUS-SimPat is implemented in German)

The *workspace* acts as start page of CASEPLUS-SimPat and provides users – family doctors or hospital employees – a general overview of their patients (Fig. 2). Here, health care professionals can access a list of their patients. In addition to a unique patient code automatically generated by the case management system, the most important master data (name, date of birth, sex) as well as case data with regard to the inpatient stay of patients (admission date, discharge date) are displayed.

#### Information Provision Modules

The primary goal of CASEPLUS-SimPat is to make information available to all actors involved in the treatment. For this purpose, the modules *case file* and *care process* were implemented.

**Fig. 3:**
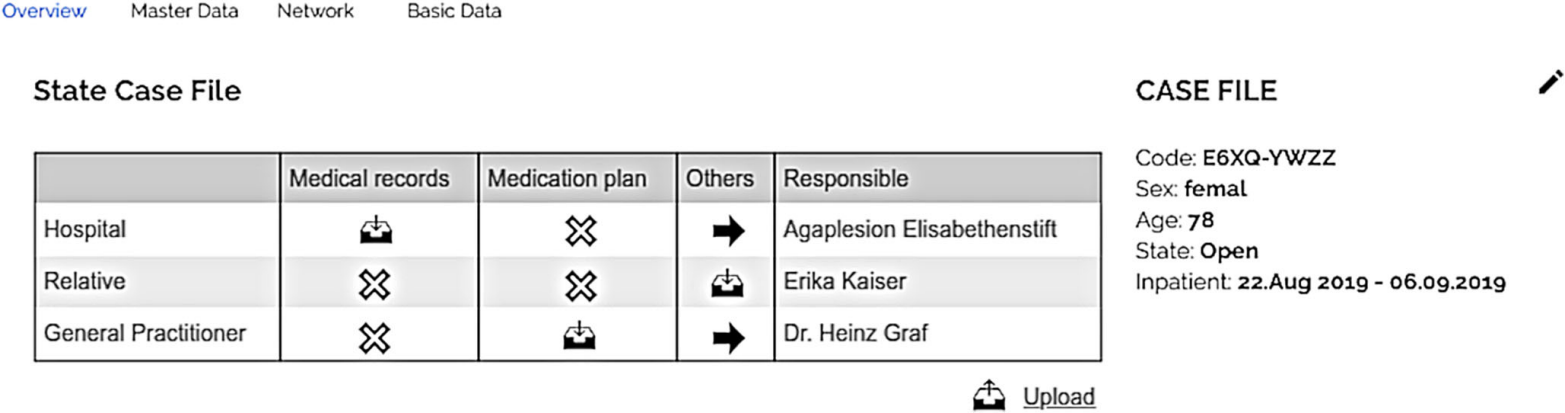
CASEPLUS-SimPat MockUp – Overview (list of organizations/persons involved and overview of uploaded documents)

The *case file* contains information about the current episode of care. Following the modular structure of CASEPLUS-SimPat, the case file is organized in four tabs: (a) overview, (b) master data, (c) basic data, and (d) network of care. The *overview* allows users to access the status of a case file. A table shows which organizations and/or persons are involved in the treatment and which documents have already been uploaded by a specific organization (Fig. 3).

The *overview* also includes a journal. It serves as central communication platform. In addition to information or questions in free text format, users can add one or more keywords to their contribution for easy retrievability (tagging). Moreover, the *master data* tab provides access to both general personal data, like name, date of birth, sex, and the health insurance number as well as to the contact details of a patient. This information is supplemented by the tab *basic data*. Here, information on the current episode (beginning, end, status quo) as well as on the inpatient stay (admission date, expected and/or definitive discharge date) is documented. Another central component is the *network of care*. It lists all known involved actors. Thereby, a distinction is made between the professional and personal network. While the professional network lists all health care professionals of a patient, the personal network documents the patient’s relatives. Hence, information about a contact person is available at a glance. This enables professional caregivers to obtain relevant information, e.g. about the medical history or allergens, as well as about the personal environment, and patient’s preferences more quickly.

The module *care process* provides a collective overview of documents uploaded for a patient during the episode of care. All documents uploaded for an organization and/or person are displayed in tabular form. To facilitate the search and selection of individual documents, all documents are grouped by actors, e.g. relatives, hospital or general practitioner. In addition, corresponding information about responsible persons and the date of first participation are included. Selected documents can be downloaded, viewed and/or saved locally.

#### Portal for Caregiving Relatives

The portal for relatives is an independent web-application as supplement to CASEPLUS-SimPat. To ensure data protection, access to CASEPLUS-SimPat and the data stored therein is not possible, as the portal is implemented separately from it.

The overall aim of the portal is to integrate relatives more closely into the care process. For this purpose, it offers possibilities to check basic information about the hospital stay and the episode (*case file*), upload or download documents (*my documents*), and access information about practitioners (*treatment process*).

**Fig. 4:**
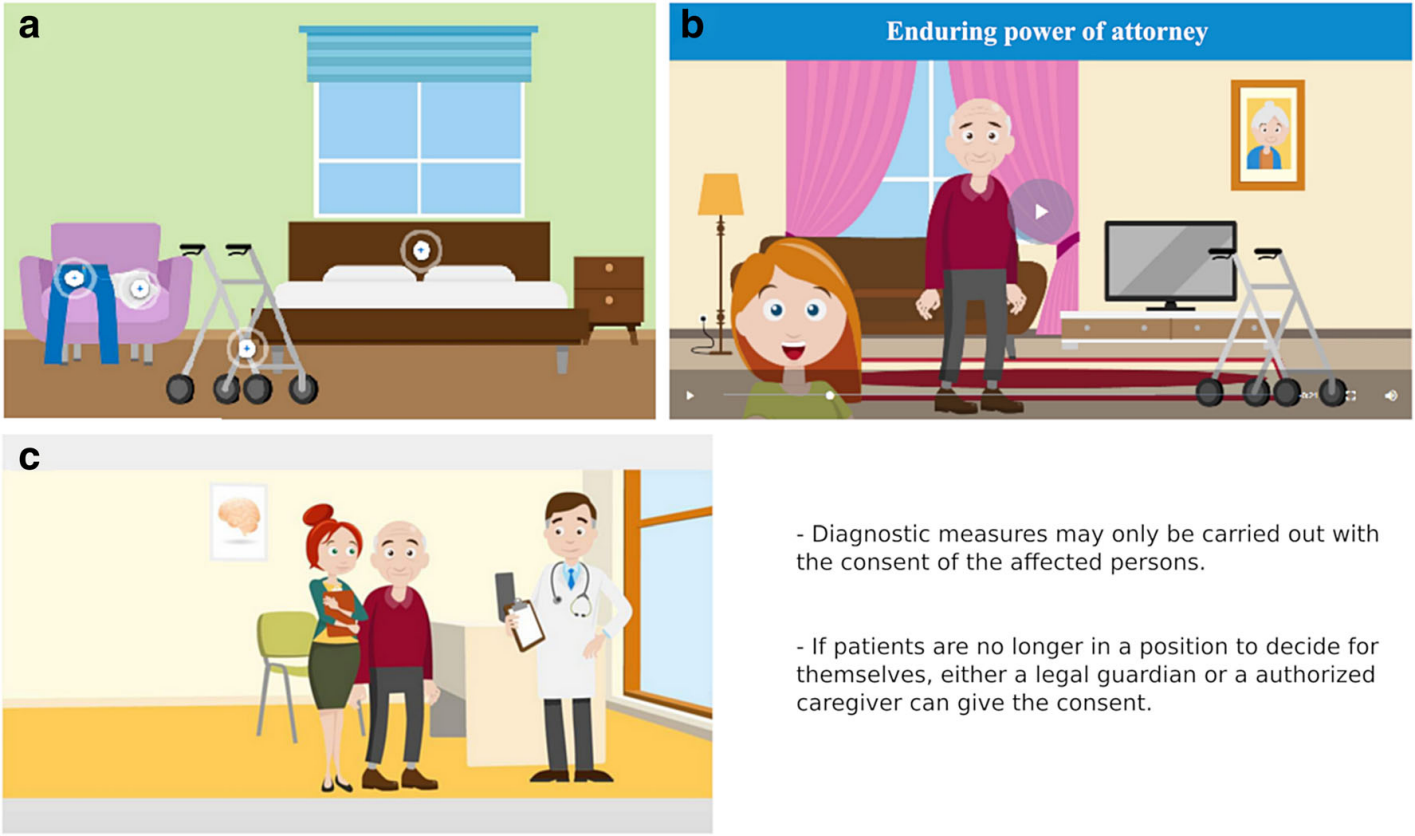
Example e-learning services within the relative’s portal. (A) haptic learning content on the topic fall prevention, (B) auditory learning content on the topic enduring power of attorney, and (C) visual learning content on the topic diagnostic

The module *courses* forms the core of the portal. General information about dementia are provided in an understandable way by means of various e-learnings (Fig. 4) to support relatives in dealing with dementia patients. Step by step, relatives are gaining knowledge about various topics:

1. Dementia basics
2. Life, handling, everyday life
3. Nutrition
4. Prevention of falls
5. Relief for relatives
6. Information during the hospital stay
7. Measures after discharge
8. Legal basics

## Usability Test

In a first evaluation step, we evaluated the usability of CASEPLUS-SimPat. We designed a set of tasks to assess the effectiveness and efficiency of CASEPLUS-SimPat. Participants had to use CASEPLUS-SimPat to solve these tasks. Additional quality criteria were used to assess the suitability for use from point of view of end users. They include criteria such as task adequacy, self-descriptiveness, expectation conformity, learning facilitation, controllability, fault tolerance, and customizability. For this, all participants completed the standardized questionnaire based on DIN ISO 9241-110 (dialogue principles) after the test-session [9].

### Participants

The usability test was conducted with future users of CASEPLUS-SimPat: two hospital employees (a social worker and a nurse of geriatrics) from EKE, and one specialist familiar with the care of dementia patients during their daily work.

The testing took place at the workplaces of the participants, ensuring that the test environment corresponds to the work environment of future users.

### Method

During a one hour evaluation, we asked the participants to solve 20 tasks with CASEPLUS-SimPat. These tasks were based on pre-determined use scenarios, for example “first login“, “search contact details”, or “search and download documents”. During the tests, the thinking aloud method was used: participants were asked to continuously express their thoughts while using the system [10]. This provides insights into the problem-solving behavior of participants and into the problems encountered when working on the tasks, allowing to understand clearly the test person’s actions. Thinking aloud protocols were used to document the thoughts of participants according to the description, influence, persistence, manifestation, and frequency of problems as well as solutions found and additional comments (see supplemental material).

### Results

The usability tests showed overall satisfaction of participants with the functionalities of CASEPLUS-SimPat. There were, however, some issues in using the software, e.g., when logging in initially or searching for specific documents. Nevertheless, all participants confirmed the importance of CASEPLUS-SimPat to ensure the cooperation of different actors within the care process.

Throughout the tests, participants provided valuable suggestions for improvement, among other things, on *sequential work steps*, *keywords used to structure journal entries* or *terminology in the user interface*. Adapting these aspects may make it even easier to find required information, and thus will increase usability. That will support the future use of the software by all involved actors in routine care.

## Conclusion and Future Work

In this paper, we presented CASEPLUS-SimPat, an intersectoral web-based case management system for multimorbid dementia patients. CASEPLUS-SimPat allows different health care professionals, hospital employees and general practitioners, to jointly access important patient data and exchange information about the current or future treatment. Better provision of structured information relevant to care thus results in improved communication and data exchange between all actors, finally benefiting patients. In addition, the supplementary portal creates opportunities for caregiving relatives to become more integrated in the care process and to receive targeted assistance through e-learnings.

In summary, CASEPLUS-SimPat allows for improved intersectoral case management. Due to the modular design and the role-based authorization concept, the case management system is easily expandable to include further functionalities for individual groups of caregivers, such as inpatient or outpatient nurses. Here, single views can be individually adapted to each group. Furthermore, by using interfaces based on interoperability standards and a web-service implementation both integration of additional care facilities and adaptability to other sites is given.

Future work within SimPat includes integrating further health care professionals and care facilities as well as testing the system in practice for its feasibility and effectiveness. Moreover, CASEPLUS-SimPat will be supplemented by new modules to provide functionalities with regard to monitoring and quality management purposes. For instance, recommendations and reminders, both for relatives and caregivers, will be used to determine whether recommended or prescribed therapeutic measures, medical checks or health aides have actually been followed after discharge from hospital.

## Electronic supplementary material

ESM 1 (DOCX 94 kb)

ESM 2 (DOCX 95 kb)

## Abbreviations

ADT: Admit Discharge Transfer
EKE: AGAPLESION ELISABETHENSTIFT
CIS: Clinical Information System
HIS: Hospital Information System
ICT: Information and Communication Technologies
MDM: Medical Document Management
SimPat: Securing Integrated Care for Multi-morbid Patients with Dementia using an IT-based Service Concept
UI: User Interface

## Footnotes

1 Forwarding to CASEPLUS-SimPat: http://www.simpat-demenz.de/
