# Supplementary material for: CASEPLUS-SimPat: An Intersectoral Web-Based Case Management System for Multimorbid Dementia Patients
Source: J Med Syst. 2020 Feb 8;44(3):63. doi: 10.1007/s10916-020-1533-9 (PMC7007915; doi:10.1007/s10916-020-1533-9)
Supplement: Supplementary file 1 — (DOCX 94 kb) [file 10916_2020_1533_MOESM1_ESM.docx]

**Usability test 2.0 – Task list**

**Software: CASEPLUS-SimPat**

Thank you for taking the time to test the case management software *CASEPLUS-SimPat*. In the following you will find some tasks to solve. Please comment on the steps you take while solving each task.

1. Please register with CASEPLUS-SimPat. (New access)
2. Now you see the list of your patients. Please call up the treatment file of “Hilde Richter”.
3. You are missing information and want to contact a relative of Mrs. Richter. Please look for the daughter’s address and telephone number.
4. The daughter (Mrs. Müller) informs you about another relative who is taking care of Mrs. Richter. Please add the following information: Susanne Held, Tel: 030 xxxxxx, Liebauer Str. 11, 10245 Berlin, can be reached well in the evening between 18-22 o’clock; niece
5. Mrs. Held will also have access to CASEPLUS-SimPat. Please set this up.
6. You notice that the attending family doctor is not yet listed in the network of Mrs. Richter. Please add this entry: Dr. Andreas Schmerz, Fichtelweg 3a, 64732 Darmstadt, 06151 xxxxxx
7. Mrs. Müller informs you of her upload of a clock-drawing test. Please search for it and download it.
8. Please find another way to download a document.
9. You have heard that Dr. Andreas Schmerz has completed a special training for the treatment of dementia patients. Please note this information.
10. Please upload a document that may be important for your relatives.
11. You accidentally uploaded the wrong document, please delete it.
12. During the last medical examination, the patient was found to be allergic to plasters. Please note this finding.
13. It turned out that especially red plasters are bad for Mrs. Richter. Please add this information to the previous one.
14. Mrs. Müller informs you that she is changing Mrs. Richter's attending physician. Please delete the current general practitioner and add the new one: Dr. Daniela Diehl
15. The new general practitioner should also be involved in the case file. Please generate a token and print it out.
16. You have received the final discharge date for the patient. Please document it in CASEPLUS-SimPat: 28.04.2019
17. Please log out.
18. Just imagine you forgot your password, please try log in again.
19. Please print out the list of your patients.
20. Please search the contact details of Dr. Annamarie André (practice Dr. André).
21. Please search the telephone number of Dr. Daniela Diehl (general practitioner at Alice Hospital).
