# Supplementary material for: CASEPLUS-SimPat: An Intersectoral Web-Based Case Management System for Multimorbid Dementia Patients
Source: J Med Syst. 2020 Feb 8;44(3):63. doi: 10.1007/s10916-020-1533-9 (PMC7007915; doi:10.1007/s10916-020-1533-9)
Supplement: Supplementary file 2 — (DOCX 95 kb) [file 10916_2020_1533_MOESM2_ESM.docx]

**Usability test 2.0 – Thinking aloud protocol (example)**

**Software: CASEPLUS-SimPat**

Explanations:

- Influence
  low = The user is briefly irritated, but quickly finds a solution without help
  medium = The user is clearly irritated and finds a solution after a long search
  high = The user is helpless and needs help
- Persistence
  low = A solution is found immediately and the user may not be aware of the issue.
  medium = The user founds a solution, but feels disturbed in fulfilling tasks.
  high = The user must always keep possible solutions in mind.
- Manifestation
  low = cosmetic problems
  medium = Problem may lead to user errors, but these are not critical
  high = The result of the issue is a user error that ends in a critical system error
- Frequency
  low = 10%, medium = 10-90%, high > 90%

**General information:**

Date: ______________________

Start of usability test: ______ End of usability test: ______ (incl. ISONORM questionnaire)

Sex:  male  female  not specified

Profession: __________________

Role/Authorization in CASEPLUS-SimPat: __________________

Has CASEPLUS-SimPat already been used?  yes  no

Keeper of the minutes: __________________

The following list contains all tasks that have been assigned to the participant. For the sake of completeness, tasks that were completed without difficulty are only listed.

# Please register with CASEPLUS-SimPat. (New access)

| Problem | Where did the problem occur? | Influence | Persistence | Manifestation | Frequency |
| --- | --- | --- | --- | --- | --- |
|  |  | low  medium  high  low  medium  high  low  medium  high | low  medium  high  low  medium  high  low  medium  high | low  medium  high  low  medium  high  low  medium  high | low  medium  high  low  medium  high  low  medium  high |
| Solutions / Annotations | | | | | |
